# Flotillins Regulate Focal Adhesions by Interacting with α-Actinin and by Influencing the Activation of Focal Adhesion Kinase

**DOI:** 10.3390/cells7040028

**Published:** 2018-04-07

**Authors:** Antje Banning, Tanja Babuke, Nina Kurrle, Melanie Meister, Mika O. Ruonala, Ritva Tikkanen

**Affiliations:** 1 Institute of Biochemistry, Medical Faculty, University of Giessen, Friedrichstrasse 24, 35392 Giessen, Germany; Antje.Banning@biochemie.med.uni-giessen.de (A.B.); Tanja.Babuke@web.de (T.B.); kurrle@med.uni-frankfurt.de (N.K.); melanie.meister@gmx.net (M.M.); 2 Department of Medicine 2, Hematology/Oncology, University Hospital, Goethe University Frankfurt, 60590 Frankfurt am Main, Germany; 3 Image Computing & Information Technologies, Kapersburgstrasse 12, 60437 Frankfurt, Germany; mika@icit.bio; 4 Center for Membrane Proteomics, University of Frankfurt, Max-von-Laue Strasse 13, 60438 Frankfurt am Main, Germany

**Keywords:** focal adhesion, cancer, metastasis, cell migration

## Abstract

Cell–matrix adhesion and cell migration are physiologically important processes that also play a major role in cancer spreading. In cultured cells, matrix adhesion depends on integrin-containing contacts such as focal adhesions. Flotillin-1 and flotillin-2 are frequently overexpressed in cancers and are associated with poor survival. Our previous studies have revealed a role for flotillin-2 in cell–matrix adhesion and in the regulation of the actin cytoskeleton. We here show that flotillins are important for cell migration in a wound healing assay and influence the morphology and dynamics of focal adhesions. Furthermore, anchorage-independent growth in soft agar is enhanced by flotillins. In the absence of flotillins, especially flotillin-2, phosphorylation of focal adhesion kinase and extracellularly regulated kinase is diminished. Flotillins interact with α-actinin, a major regulator of focal adhesion dynamics. These findings are important for understanding the molecular mechanisms of how flotillin overexpression in cancers may affect cell migration and, especially, enhance metastasis formation.

## 1. Introduction

Cell migration is a crucial process in, e.g., embryonic development, tissue repair and cancer. Migration is a multistep process, basically comprising the formation of protrusions, the generation of cell–matrix adhesions, the translocation of the cell body, the release of adhesions and, finally, the retraction of the cell rear, thus requiring a precise spatial and temporal regulation [1,2,3]. After protrusions have formed, they need to be stabilized by connections to the extracellular matrix (ECM). These cell–matrix adhesions are classified into focal complexes, focal adhesions, fibrillar adhesions (also called focal contacts), and podosomes [4]. The dot-like, small focal complexes lie at the tip of lamellipodia and form a first contact between the ECM and the cell. During migration, they can undergo maturation into focal adhesions. Since the precise discrimination between these three forms of adhesions is sometimes difficult, the term “focal adhesion” (FA) is often used for all adhesion types, also in this paper. 

Cell spreading and cell migration are in many ways analogous processes, as they both depend on the formation of FAs. However, during cell spreading after detachment, the cell needs to generate cell–matrix adhesions *de novo*. Cell spreading is therefore an ideal tool to investigate the formation of cell–matrix adhesions. In addition, signaling events (especially Tyr kinase activities) upon substrate attachment and cell spreading can be analyzed with biochemical methods [5,6]. 

Integrins are transmembrane receptors that bind molecules of the extracellular matrix with their N-terminal domain and cytoskeleton associated proteins via their cytoplasmic C-terminus. Upon integrin activation, FA proteins are sequentially recruited to cell–matrix adhesions. One of the first major signaling events is the auto-phosphorylation of focal adhesion kinase (FAK), which results in the formation of the FAK–c-Src complex. This complex then phosphorylates and recruits further proteins, establishing a connection to the actin cytoskeleton (Reviewed in [2,7]).

Upon activation and clustering of integrins, many of the recruited proteins primarily bind to the cytoplasmic integrin β-subunit tail. Some proteins serve as a direct link to the actin cytoskeleton, such as α-actinin and filamin. Others, such as FAK, c-Src, and paxillin, are signaling components that help to recruit more proteins and to activate downstream signaling [7,8,9]. Paxillin directly binds to integrin β-subunit and is necessary for the recruitment of FAK to cell–matrix adhesions. FAK is a tyrosine kinase representing one of the major scaffolding and signaling molecules in FA formation. Upon FA remodeling, autophosphorylation of Tyr 397 in FAK creates a binding site for c-Src, which phosphorylates FAK, thereby increasing its kinase activity [10]. The resulting FAK–c-Src complex is crucial for further phosphorylation and recruitment events within FAs. Src-dependent phosphorylation of Tyr 925 of FAK creates a binding site for Grb2 (growth factor receptor-bound protein 2), which activates ERK (extracellularly regulated kinase) via the conventional Ras/MEK pathway [11]. ERK2 influences FA regulation and is connected with proliferation and survival signals within the cell. A large number of proteins have been localized to FAs, and yet further ones have been shown to influence FAs [7]. Thus, the regulation of FAs and their dynamics during cell–matrix adhesion and migration is a very complex process that involves numerous players, some of which still remain to be identified. 

The family of flotillin proteins contains two well-conserved, ubiquitously expressed members, flotillin-1 and flotillin-2, also called reggie-2 and reggie-1, respectively (for a review, see [12,13,14]). Flotillins have been shown to be associated with detergent-resistant membranes or membrane rafts by means of palmitoylation (both flotillins) and myristoylation (only flotillin-2) [15,16]. They exhibit a strong tendency to form both homo- and heterooligomers, which regulate their cellular localization and function [17,18]. Flotillins have been shown to be associated with several signal transduction pathways, especially mitogen-activated protein kinases (MAPK) [19,20], and to be Tyr phosphorylated by the Src kinase family members [17,21,22]. In addition, a role in membrane trafficking and especially endocytosis has been suggested for flotillin-1 [14,23,24,25,26]. Flotillins are also involved in cell–cell adhesion, and they regulate both adherens junctions and desmosomes [27,28,29]. Although flotillins are involved in many important cellular processes, they do not appear to be essential either in invertebrates [30] or in vertebrates, as evidenced by a lack of any severe phenotype in flotillin-1 and flotillin-2 knockout mice [31,32,33]. 

We have previously shown that flotillin-2 may function in cell–matrix adhesion during cell spreading, since overexpression of flotillin-2 accelerates cell spreading, whereas its knockdown inhibits it [17,21]. Furthermore, Tyr phosphorylation of specific residues appears to be important for the function of flotillin-2 in cell spreading [21], and overexpression of flotillin-2 results in numerous cell protrusions, indicating a role in actin remodeling [16,17,21,34]. A possible connection to the actin cytoskeleton and cell adhesion is established by the interaction of flotillins with the members of the Sorbin homology protein family (reviewed in [35]), which have been shown to regulate actin-dependent processes and to be involved in cell adhesion [36,37]. By means of their interaction with the Cbl-associated protein (CAP), flotillins have been suggested to regulate cytoskeletal remodeling during neuronal differentiation and to affect the activation of the Rho family of GTPases [38,39]. Flotillin-1 also appears to play a role in cell polarity during migration, since flotillin-1-deficient neutrophils display severe problems in forming a polarized tail [33]. In line with a function during cell migration, flotillins have even been suggested to localize to FAs together with vinculin and CAP in neuronal cells seeded on laminin, and a mild effect on FAK phosphorylation upon flotillin-2 knockdown was reported after IGF-1 stimulation of N2a cells [39,40]. We have shown that flotillin-1 and CAP both directly interact with Fibroblast Growth Factor Receptor Substrate 2 (FRS2) [41]. However, the molecular details of the role of flotillins in cell–matrix adhesion still remain an open question.

In this study, we aimed at characterizing the molecular mechanisms of flotillin function in cell–matrix adhesion and the role of flotillins in the regulation of focal adhesions. We here show that flotillins are important for cell migration and morphology of focal adhesions. Small-interfering RNAs (siRNAs) against flotillin-2, but not flotillin-1, affect the autophosphorylation of FAK on Tyr397, indicating a role in the regulation of FA dynamics, whereas siRNAs against both flotillins impair ERK phosphorylation upon cell spreading. We identify α-actinin as a direct interaction partner of flotillins, which may provide a molecular link between flotillins, FAK and the regulation of FA dynamics. Considering the established role of flotillins in cancer and metastasis formation, our findings are also relevant for understanding cancer spreading. 

## 2. Materials and Methods

### 2.1. Cell Culture, Transfection, and RNA Interference

HeLa cells were cultured in Dulbecco’s Modified Eagle’s Medium (DMEM) with 10% fetal calf serum (FCS; Invitrogen, Thermo Fisher Scientific, Karlsruhe, Germany) at 37°C under 8% CO_2_. MCF10A cells were cultivated in DMEM/F-12 (1:1) with 3% FCS and 2% horse serum (Invitrogen), 1% penicillin/streptomycin, epidermal growth factor (20 ng/mL), dexamethasone (0.4 µg/mL, Sigma-Aldrich, Taufkirchen, Germany), cholera toxin (100 ng/mL, Sigma-Aldrich), and insulin (10 µg/mL, Sanofi Aventis, Frankfurt am Main, Germany). Transfections of HeLa cells were performed using Lipofectamine2000 (Invitrogen). The flotillin siRNAs (Stealth siRNA, Invitrogen) have been described previously [17,21,31]. Flotillin-1–EYFP and –EGFP were obtained from D. Browman [42]. The coding regions of rat flotillin-2 and human α-actinin 1 were cloned into pEYFP-N1 and pECFP-N1, respectively, and in pGEX-4T-1 (α-actinin 1). His–α-actinin has been described before [43]. The stable flotillin knockdown MCF10A cell lines have been described previously [17,44]. 

### 2.2. Antibodies

Flotillin-1 and flotillin-2: mouse monoclonal (BD Biosciences, Heidelberg, Germany) and rabbit polyclonal (Sigma-Aldrich) antibodies; vinculin: mouse monoclonal antibody (Sigma-Aldrich); FAK and pY397-FAK: mouse monoclonal (BD Biosciences); ERK1/2: rabbit polyclonal (C-14, Santa Cruz Biotechnology, Heidelberg, Germany) antibodies; pERK2: mouse monoclonal antibody (E-4, Santa Cruz); GAPDH: mouse monoclonal antibody (Abcam, Cambridge, UK); α-actinin: rabbit polyclonal H-300 antibody (Santa Cruz) for IP, mouse monoclonal antibody (BD Biosciences) for Western blot and mouse monoclonal antibody (BM-75-1, Sigma-Aldrich) for immunofluorescence; His-tag: mouse monoclonal antibody (Novagen, Darmstadt, Germany), myc-tag: mouse monoclonal antibody (Santa Cruz Biotechnology).

### 2.3. Immunofluorescence 

Cells cultured on coverslips were fixed with 4% PFA, followed by permeabilization with 50 µg/mL digitonin (for spreading assays and staining of FAs) or with MeOH at −20 °C (for staining of flotillins). Thereafter, the cells were incubated with the primary antibody in 1% BSA or Alexa Fluor 488-conjugated phalloidin (Molecular Probes, Eugene, OR, USA), washed, incubated with the secondary antibody and mounted in GelMount (Biomeda, Foster City, CA, USA). The samples were analyzed with a Zeiss LSM 510 or LSM 710 Confocal Laser Scanning Microscopes (Carl Zeiss, Oberkochen, Germany).

To visualize FAs, the coverslips were coated with either collagen or fibronectin (both 10 µg/mL). The number of FAs was determined in HeLa cells seeded on collagen. For counting FAs, the size of the cells was determined by measuring their length and width. Focal adhesions were counted only in cells exhibiting a certain size range (mean of each experiment ±25%). The experiment was repeated five times, and 50 cells were analyzed in total for each condition.

### 2.4. Cell Lysis, Gel Electrophoresis, and Western Blot 

The cell pellets were lysed in lysis buffer (50 mM Tris pH 7.4, 150 mM NaCl, 2 mM EDTA, 1% Nonidet P-40), and the lysates were cleared by centrifugation. When phosphorylated epitopes were analyzed, the cells were lysed in phospholysis buffer (100 mM Tris pH 8.0, 150 mM NaCl, 1 mM MgCl_2_, 1% Triton X-100) supplemented with 1 mM Na-vanadate. The protein concentration was measured with the Bio-Rad Protein assay reagent (Bio-Rad, Munich, Germany). Equal amounts of the lysates were analyzed by SDS-PAGE and Western blot. The Quantity One program (Bio-Rad) was used for densitometric quantification of the Western blot signals.

### 2.5. Immunoprecipitation

MF10A cells were lysed for 30 min on ice in immunoprecipitation lysis buffer (10 mM Tris-HCl, pH 8.0, 150 mM NaCl, 5 mM EDTA, 0.5% Triton X-100, and 60 mM N-octylglucoside) supplemented with protease inhibitors and cleared by centrifugation. Unspecific binding material was removed by incubating the lysates with Pansorbin beads (Calbiochem, Darmstadt, Germany). The polyclonal anti-α-actinin antibody was precoupled with Dynabeads Protein A (Invitrogen) and combined with an amount of lysate corresponding to 500 µg of total proteins. Mock-incubated beads and MCF10A cell lysates were used for control precipitation. 

### 2.6. GST Pulldown Assay

Indirect GST pulldown assays were performed essentially as described in Völlner et al. [29]. Briefly, pulldowns were carried out with 5 μg α-actinin-1–GST coupled to glutathione sepharose beads with MCF10A control, flotillin-2 shRNA, and flotillin-1 shRNA cell lysates. For each indirect pulldown, cell lysates containing 1.2 mg of total protein were used. GST (expressed from pGEX-4T1) was used as a control. 

### 2.7. Förster Resonance Energy Transfer- Fluorescence Lifetime Imaging (FRET–FLIM) 

HeLa cells were transfected with Lipofectamine2000, transferred to coverslips and fixed 2 days later with PFA, followed by quenching with 50 mM Gly/PBS and embedding in Mowiol. The fluorescence lifetime (tau, τ) of the FRET donor was determined in a Time-Correlated Single Photon Counting unit (TCSPC, Becker&Hickle GmbH, Berlin, Germany) connected to a Leica DM IRE2 AOBS confocal microscope (Leica Microsystems, Heidelberg, Germany). The donor fluorochrome (ECFP) was excited with a Coherent MIRA (Coherent, Göttingen, Germany) pulse intensified femtosecond two-photon laser set at 820 nm, and the emission signal was typically recorded for 10 min through a 480/30 BP filter. For the details of the data analysis and statistical calculations, please refer to Babuke et al. [17].

### 2.8. Cell Spreading Assay 

Chamber Slides (Lab-TekII Chamber German Coverglass System with 4 chambers) were coated with fibronectin, followed by blocking with 0.5% BSA/PBS. The siRNA-transfected cells were detached with 0.05% EDTA/PBS, and 40,000 cells were seeded per chamber in a medium containing FCS and incubated at 37°C. After 25 min, the cells were fixed with 4% PFA and stained with Alexa Fluor 488-conjugated phalloidin. The chamber slides were mounted in GelMount and further analyzed with the Zeiss LSM510 Confocal Laser Scanning Microscope (Carl Zeiss, Oberkochen, Germany). The cells were classified as non-spread, half-spread, or completely spread. For the exact classification criteria, please refer to [21]. For the analysis of phosphorylation during spreading, the cells were starved for 24 h, and the experiment was performed without serum. The cells were detached as above and resuspended in DMEM with 10 mM Hepes, pH 7.4. The cell suspension was kept rolling at 37°C for 15 min, and the cells were directly plated on a fibronectin-coated surface (coverslips or 6 cm dishes) and kept at 37 °C for the indicated time points. 

### 2.9. Haptotactic Cell Migration Assay

The lower side of Polycarbonate Membrane Transwell^®^ Inserts with 8 µm pore size on a 24-well plate (Corning, Corning, NY, USA) was coated with fibronectin (10 µg/mL in PBS, 30 min). The inserts were washed and placed in migration medium (DMEM with 1 mM MgCl_2_ and 0.5% BSA) overnight. Fresh medium was added the next day. The haptotactic migration assay was carried out 3 days post-siRNA transfection by seeding 25,000 cells per well into the upper chamber. After 6 h, the membranes were washed with PBS, fixed, and stained with crystal violet (2.3% in 20% Ethanol; Sigma-Aldrich) with subsequent extensive washing. The staining in the upper part was removed with a Q-tip, and the membranes were dried overnight and then destained with 10% acetic acid. The eluted stain was measured at 595 nm, and the measured values, which are directly proportional to the number of migrated cells, were correlated to those corresponding to the control cells.

### 2.10. Wound Healing

The migration of flotillin knockdown cells was measured using the wound healing assay. HeLa cells were grown to confluence in 6-well plates. In confluent monolayers, a wound was made with a pipette tip, followed by extensive washing with PBS to remove the cell debris. The cells were then cultured in DMEM containing 2% FCS and allowed to migrate into the wound area for 24 h at 37 °C. At different time points, the wound closure was photographed and analyzed with ImageJ software (NIH, Bethesda, MD, USA). To exclude a proliferation effect, Mitomycin C (10 µg/mL; Sigma-Aldrich) was added to the medium during wound closure. At the end of the migration period, the cells were harvested and used for Western blot analyses or, after propidium iodide staining, for fluorescence-associated cell sorting. 

### 2.11. Cell Growth in Soft Agar

Flotillin knockdown or overexpressing cells (1 × 10^4^ cells) were diluted in 1 mL premix (670 µL Hela-conditioned DMEM, 200 µL 6xDMEM, 130 µL FCS) and mixed with 1 mL of warm 0.7% bacto-agar (Becton Dickinson, Heidelberg, Germany). The suspension was overlaid onto a 2 mL solidified bottom layer of premix containing 1% bacto agar in 6-well plates. The plates were incubated at 37°C in 8% CO_2_. Once a week, 200 µL DMEM with 10% FCS was added. After 2 weeks, colony formation was visualized by staining with 0.01% crystal violet. The plates were analyzed with a Leica MZ16 stereomicroscope, and the colonies were counted with the GSA image analyzer software (GSA, Rostock, Germany).

### 2.12. Statistical Analysis and Quantification

At least three independent experiments were performed for each assay. Shown is the mean ± SD. Densitometric quantification was performed with the Quantity One Program (Bio-Rad). Statistical analysis (Anova tests) was done with the GraphPad Prism 5 program (GraphPad, La Jolla, CA, USA). One-way Anova with the Dunnett’s post-test was used to compare each treatment (different siRNAs) with the control. Thus, *p*-values represent the statistical significance of the differences between treatment and control conditions. In some cases, *t*-test or Two-way Anova with Bonferroni’s post-test were performed. The significance of each value is shown with respect to the corresponding control values. The *p*-values are defined as follows: * *p* < 0.05; ** *p* < 0.01, *** *p* < 0.001.

## 3. Results

### 3.1. Flotillin Knockdown Impairs Cell Migration and Spreading

Our earlier studies have shown that overexpression of flotillin-2 accelerates, and its depletion inhibits cell spreading on fibronectin [21], suggesting that flotillin-2 is important for the regulation of focal adhesions, which are integrin-based cell–matrix adhesion structures. However, since depletion of flotillin-2 also results in severely reduced expression of flotillin-1 in many cell lines and in the knockout mouse models [13,31,33,44], it has not been possible to directly identify the specific role of each flotillin in adhesion. Thus, it was important to test if siRNAs against flotillin-1, which reduce but do not completely ablate the expression of flotillin-2, would affect cell–matrix adhesion and cell migration. In all RNAi-based assays used in this paper, we generally obtained a knockdown of flotillins of about 90% at the protein level by using two different, well-characterized siRNA sequences [17,19,21,31,44] directed against each flotillin in HeLa cells (Supplementary Figure S1a). Flotillin-2 knockdown resulted in about 85% depletion of flotillin-1 as well, whereas flotillin-1 knockdown reduced the levels of flotillin-2 to about 50% (Supplementary Figure S1b). 

**Figure 1:**
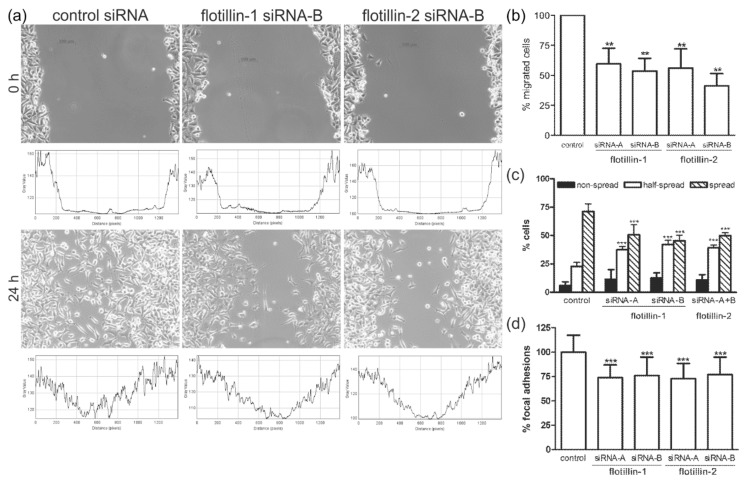
Flotillin knockdown cells display a reduced migration rate in a wound healing assay, and depletion of flotillins results in impaired haptotactic migration, slower cell spreading and reduced number of FAs. (**a**) HeLa cells transfected with the indicated siRNAs were allowed to grow until confluent. A defined scratch was then produced (0 h, upper panels), and the closure of the wounded area was monitored over 24 h (lower panels). The photographs show a representative section from *n* ≥ 3 experiments. The graphs represent plot profiles with integrated pixel density across the wound area. (**b**) HeLa cells were transfected with the indicated siRNAs. The lower side of a Transwell membrane was coated with fibronectin, and the cells were seeded in the upper part. After 6 h, the amount of migrated cells on the lower membrane part was measured. The control siRNA sample was used as the reference value and set to 100%. At least five independent experiments with duplicates per sample were performed (*n* ≥ 5, ** *p* < 0.001; One-way Anova). (**c**) HeLa cells were transfected with the indicated siRNAs, detached, and then seeded on fibronectin for 25 min. The cells were morphometrically scored as non-spread, half-spread, or spread. At least 200 cells were counted for each sample in at least four independent experiments. For flotillin-2, the results with the two siRNAs were combined. The bars show mean ± SD (*n* ≥ 4, *** *p* < 0.001, Two-way Anova, significance is shown against the corresponding control value). (**d**) HeLa cells were transfected with the indicated siRNAs, focal adhesions were visualized by vinculin staining, and their number per cell was determined. For counting, the size of the cells was measured, and only cells within a certain size range (±25% of average within each experiment) were analyzed to avoid bias due to heterogeneous cell size. At least 50 cells per sample were counted. The mean of the control sample was used as the reference value and set to 100%. At least five individual experiments were performed. The bar graphs represent the mean ± SD (*** *p* < 0.001; One-way Anova).

To analyze the migration of flotillin siRNA-transfected cells, we used a wound healing assay in which a monolayer of cells is damaged by producing a scratch of a standard width, and the closing of this wound by cells migrating towards each other from both sides is monitored. After 24 h, control siRNA-transfected HeLa cells had closed the wound, whereas with flotillin-1 or flotillin-2 siRNA-transfected cells, an open space between the wound edges was still observed (Figure 1a). To exclude the effect of possible proliferation differences on the results, we performed the experiment under Mitomycin C treatment with virtually identical results (Supplementary Figure S1c,d). The effect of Mitomycin C treatment on the cell cycle is shown in Supplementary Figure S1e. These data suggest that cell migration is impaired upon ablation of flotillins. 

Since the wound healing assay is not capable of distinguishing between a slower forward migration speed and an increased random, non-directional migration, which both would result in delayed closing of the wound, we used a haptotactic Boyden chamber assay to measure cell migration towards fibronectin as a chemoattractant (Figure 1b). After 6 h, significantly less flotillin-1 and flotillin-2 knockdown cells had migrated towards fibronectin as compared to control siRNA-transfected cells. The results for all four flotillin siRNAs used showed a statistically highly significant (*p* < 0.001) difference to the controls.

To measure the dynamics of focal adhesions, we used a cell spreading assay that we have previously established [21,37], which morphometrically measures the degree of cell spreading on fibronectin-coated surfaces (Figure 1c). Knockdown of flotillin-1 by means of two different siRNAs resulted in significantly decelerated spreading, manifested as a higher fraction of incompletely spread cells. This effect was comparable to that of flotillin-2 siRNAs, the results of which were in accordance with the data previously published by us [21] and are thus shown as a summary of both flotillin-2 siRNAs in Figure 1c. 

Taken together, the above results would suggest that both flotillins are involved in the regulation of cell–matrix adhesion and cell migration, and their relative expression levels may also impact on cell–matrix adhesion. FAs are integrin-dependent adhesion points required for cell migration and spreading. Since both processes were affected by flotillin depletion, we analyzed the effect of flotillin knockdown on the number and morphology of FAs in steady-state cells that had adhered for 24 h. Supplementary Figure S2 shows the corresponding vinculin staining of FAs in control and flotillin knockdown cells. The number of FAs in flotillin knockdown cells was found to be highly significantly reduced (by about 25%) in the fully spread cells (Figure 1d).

### 3.2. Flotillin Expression Enhances Anchorage-Independent Growth

**Figure 2:**
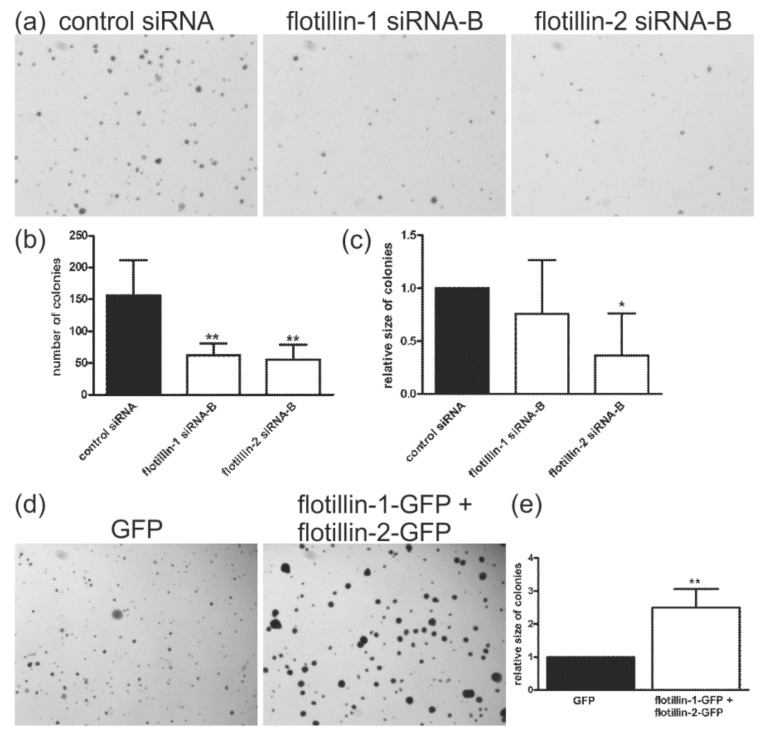
Knockdown of flotillins reduces anchorage-independent growth in soft agar, whereas overexpression results in larger colonies. (**a**) HeLa cells transfected with the indicated siRNAs were seeded into soft agar and allowed to grow for 14 days, after which the colonies were counted (**b**), and their average size determined (**c**). The bars show mean ± SD (*n* ≥ 4, * *p* < 0.05, One-way Anova). (**d**) HeLa cells were transfected with the indicated constructs and then processed as in (**a**). (**e**) Quantification of the data shown in (**d**). The bars show mean ± SD (*n* = 5, ** *p* < 0.01, *t*-test, significance is shown against the corresponding control value).

Migration of tumor cells is a prerequisite for the formation of metastases, and flotillin-2 knockout has been shown to impair metastasis formation in a breast cancer mouse model [32]. We reasoned that flotillin knockdown might display an inhibitory effect on the anchorage-independent growth of cancer cells in a soft agar assay, which is an *in vitro* assay for the metastatic potential of cells. We implanted siRNA-transfected cells into soft agar, and colony formation was assayed 14 days later (Figure 2a). Control siRNA-transfected cells formed significantly more colonies than the knockdown cells (Figure 2b). In addition, flotillin-2 siRNA cells showed a significantly reduced colony size (Figure 2c). Earlier findings have suggested that increased flotillin-2 expression correlates with a high metastatic potential of melanoma cells [45]. Thus, we studied the effect of overexpression of flotillins on colony formation (Figure 2d). As compared to the control cells transfected with EGFP, cells cotransfected with flotillin-1–EGFP and flotillin-2–EGFP (Figure 2d) did not show a statistically significant change in the number of colonies. However, the average size of the colonies was significantly increased in the case of flotillin-1–EGFP and flotillin-2–EGFP cotransfection, as compared to EGFP-transfected cells (Figure 2e). 

### 3.3. Flotillin Knockdown Affects Focal Adhesion Morphology

**Figure 3:**
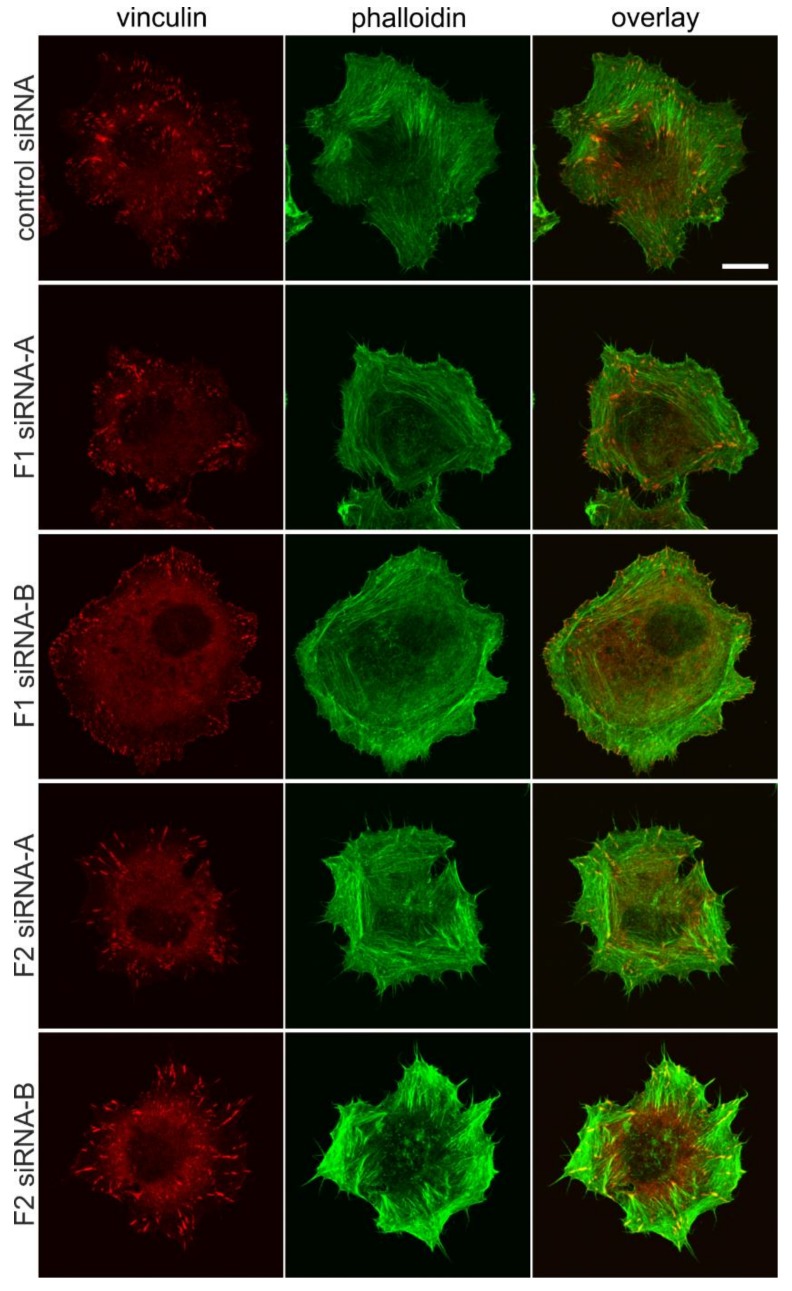
Knockdown of flotillin-1 or flotillin-2 results in altered focal adhesion morphology during cell spreading. HeLa cells were transfected with the indicated siRNAs. The cells were detached, kept in suspension, and then seeded on fibronectin for 25 min. Focal adhesions were visualized by vinculin staining and filamentous actin by phalloidin staining. Scale bar: 10 µm, the same magnification for all images.

Since the FAs in flotillin knockdown cells appeared to be also morphologically altered in fully spread cells, we studied the morphology and cellular location of FAs after spreading on fibronectin by means of vinculin and phalloidin staining. Figure 3 shows the staining of vinculin and phalloidin in HeLa cells after 25 min spreading on fibronectin. No obvious difference in vinculin staining was observed between the control cells (Figure 3, uppermost row) and flotillin-1 siRNA cells (Figure 3, rows 2 and 3). However, in flotillin-2 siRNA cells, FAs stained with vinculin appeared more elongated (Figure 3, rows 4 and 5). Furthermore, flotillin-1 siRNA cells displayed an actin cytoskeleton heavily organized as a cortical ring instead of fibers running towards the periphery, which was not observed in flotillin-2 siRNA-transfected cells. 

**Figure 4:**
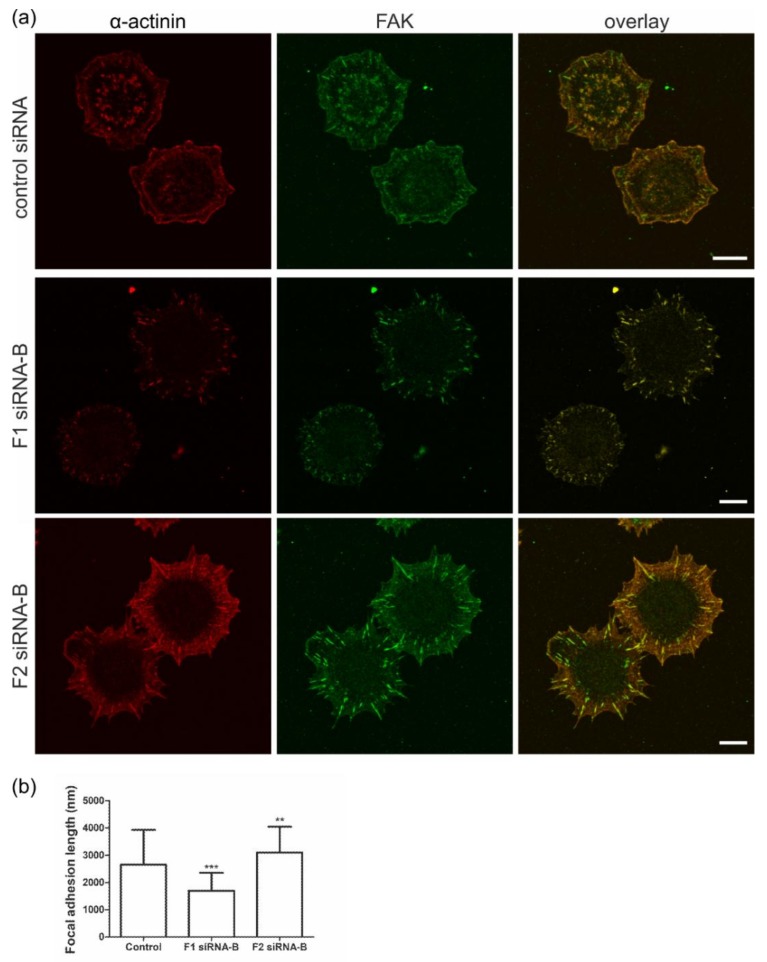
Knockdown of flotillins results in altered morphology and length of focal adhesions during cell spreading. HeLa cells were transfected with control siRNAs or siRNAs against flotillin-1 or flotillin-2. (**a**) The cells were detached, seeded for 25 min on fibronectin, and stained for α-actinin and FAK. (Scale bars = 10 µm) (**b**) The quantification of FA length was performed with Image J using FAK staining. The graph shows the length of FAs in nm. For the quantification, at least nine cells (60–180 FAs) from three individual experiments were quantified. (** *p* < 0.01, *** *p* < 0.001)

To verify these data with other FA markers, we next studied if knockdown of flotillins would alter FA morphology and length using α-actinin and FAK staining. HeLa cells transfected with control siRNA, flotillin-1 or flotillin-2 siRNAs were plated on fibronectin, allowed to adhere for 25 min and then stained for α-actinin and FAK (Figure 4a). As compared to the control cells, FAs in flotillin-1 siRNA-transfected cells appeared shorter, whereas in flotillin-2 knockdown cells, both α-actinin and FAK were colocalized in highly elongated structures at the cell periphery. The quantification of the data indeed showed that the FA length in flotillin-1 siRNA cells was significantly reduced, whereas flotillin-2 siRNA cells exhibited significantly elongated FAs. Thus, the siRNAs against flotillins display different effects on FA length, and flotillin-1 knockdown (which also reduces flotillin-2 amount) shortens FAs, whereas knockdown of flotillin-2 with concomitant flotillin-1 ablation causes elongation of FAs and concentration of α-actinin and FAK in these structures. 

### 3.4. Flotillin-2 siRNA Results in Reduced FAK Autophosphorylation

**Figure 5:**
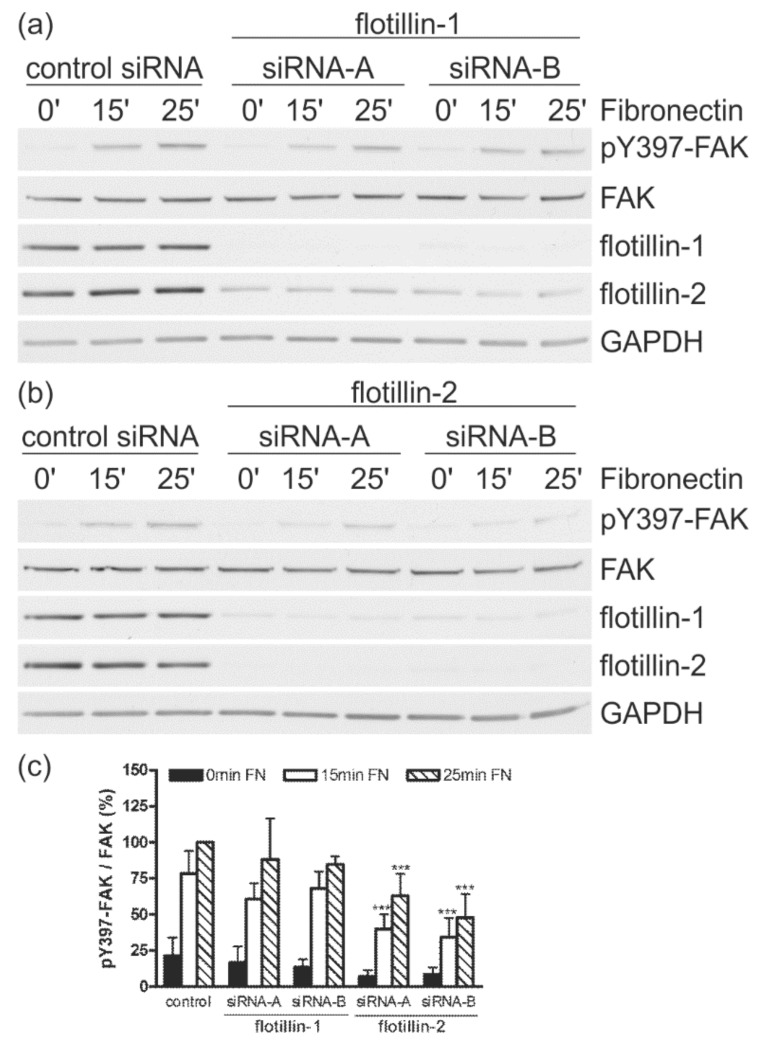
Transfection with siRNAs against flotillin-2 but not against flotillin-1 reduces autophosphorylation of FAK. HeLa cells were transfected with the indicated siRNAs. The cells were detached, kept in suspension, and then seeded on fibronectin for different times as indicated. Autophosphorylation of FAK was measured by Western blot with a phospho-site-specific antibody (pY397-FAK) in flotillin-1 (**a**) or flotillin-2 (**b**) siRNA-transfected cells. GAPDH and FAK were used as equal loading controls. (**c**) For quantification, the pY397-FAK signal in each sample was first normalized to the total FAK signal. The highest phosphorylation value (control, 25 min FN) was used as the reference and set to 100%, to which all other values were correlated. The bars represent the mean ± SD of at least four independent experiments. For statistical analysis, the significance of each value against the corresponding control value is shown (*n* ≥ 4, *** *p* < 0.001, 2-way Anova). Please note that the data in Figure 5a,b and Figure 6a,b are derived from the same experiment and thus contain identical panels for flotillins and GAPDH.

FAs are highly dynamic structures that are continuously restructured during cell spreading and migration. Focal adhesion kinase (FAK) is one of the key regulators of FA dynamics. Autophosphorylation of FAK on Tyr397 is one of the early events that take place after integrin engagement [10,11]. Thus, the phosphorylation status of FAK in Tyr397 is a good indicator of signaling upon integrin activation during spreading. Control and flotillin knockdown cells were starved, detached and then kept in suspension for 15 min to reduce basal FAK phosphorylation. The cells were then seeded on fibronectin for the indicated times, harvested, and FAK phosphorylation was analyzed with a phospho-site-specific antibody (pY397-FAK; Figure 5). Interestingly, flotillin-1 siRNA transfected cells showed similar levels of pY397-FAK as control cells (Figure 5a), whereas flotillin-2 siRNA transfection (Figure 5b) resulted in a statistically highly significant reduction in phosphorylated Y397-FAK after 15 and 25 min (Figure 5c). These data suggest that flotillin-1 and flotillin-2 siRNAs display distinguishable effects on FAs, as flotillin-1 siRNAs do not ablate FAK phosphorylation, whereas flotillin-2 siRNAs, with concomitant impairment of flotillin-1 expression, do.

### 3.5. Flotillin Depletion Impairs ERK Activation during Cell Spreading

**Figure 6:**
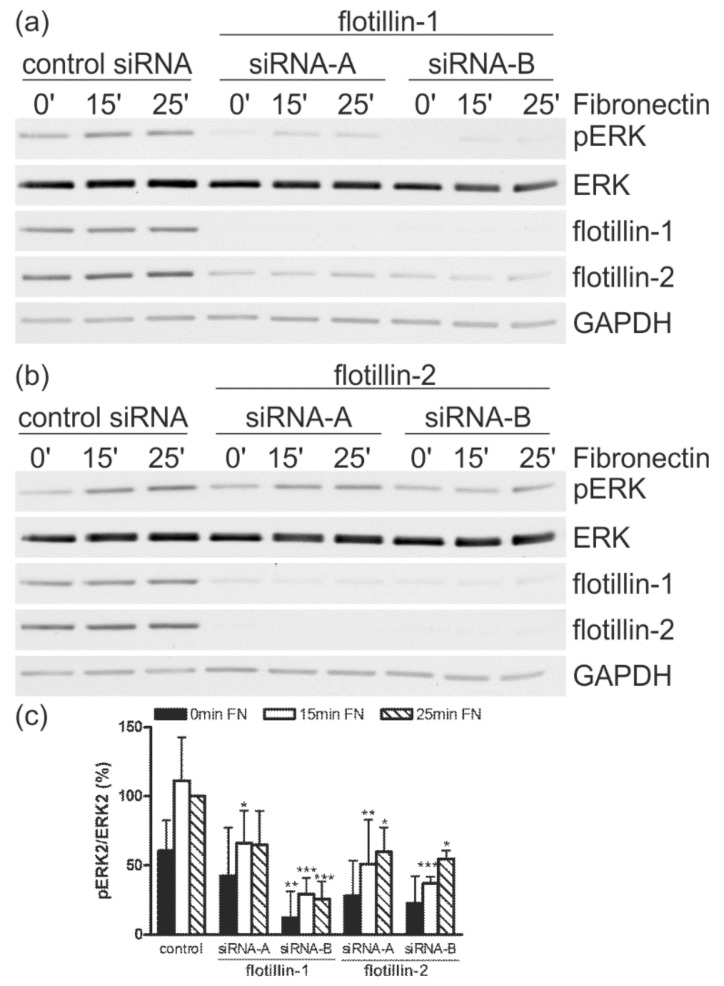
Transfection with siRNAs against flotillin-1 or flotillin-2 reduces ERK2 activation upon integrin stimulation. HeLa cells were transfected with the indicated siRNAs against (**a**) flotillin-1 or (**b**) flotillin-2, or with a control siRNA. The cells were detached, kept in suspension, and then seeded on fibronectin for the indicated time points. (**c**) For quantification, the value for the control siRNA cells (25 min) was used as a reference sample and set to 100%. The phospho- (p)ERK signal in each sample was first normalized to the total ERK signal and then correlated to the reference sample. The bars represent the mean ± SD of at least three independent experiments. For statistical analysis, the significance of each value against the corresponding control value is shown (*n* ≥ 3, * *p* < 0.05, ** *p* < 0.01, *** *p* < 0.001, Two-way Anova).

Our earlier data show that knockdown of flotillin-1 in HeLa cells impairs signaling from EGF receptor to the MAP kinase ERK, resulting in its reduced phosphorylation [19], whereas knockout of flotillin-2 in the mouse increases ERK signaling [31]. During cell–matrix adhesion, ERK also becomes phosphorylated in an event that takes place further downstream of FAK phosphorylation. We thus measured the phosphorylation of ERK during adhesion of the cells on fibronectin, using identical conditions as above. As compared to the control siRNA cells, transfection with either flotillin-1 (Figure 6a) or flotillin-2 (Figure 6b) siRNA resulted in a statistically significant reduction in the phosphorylation of ERK after 15 and 25 min adhesion, although the effect was somewhat weaker with flotillin-2 siRNAs (Figure 6c). Thus, in the context of cell spreading, all four flotillin siRNAs reduced ERK-dependent signaling.

### 3.6. Flotillins Interact with α-Actinin

**Figure 7:**
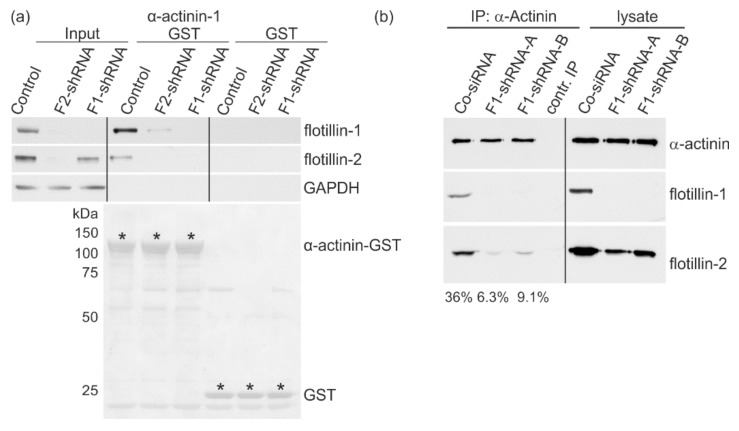
Flotillin-2 and α-actinin interaction depends on flotillin-1 expression. Stable control and flotillin-1 knockdown MCF10A cells were grown on 10 cm plates to confluence, lysed, and used for a GST pulldown or coimmunoprecipitation. (**a**) Purified recombinant human α-actinin-1–GST was used to pull down interacting proteins from MCF10A cell lysates, and GST was used as a negative control. The interaction with flotillins was analyzed by Western blot. GAPDH shows equal loading of the lysates and functioned as a negative control for the pulldown. Lowermost blot: Ponceau staining for the fusion proteins (marked with *) used in this assay. (**b**) Cell lysates were precipitated with a polyclonal antibody against α-actinin or mock-precipitated. The immunoprecipitates were analyzed for the presence of α-actinin and coimmunoprecipitated flotillin-1 and flotillin-2 by means of Western blot (*n* = 6). Flotillin-2 signals in the immunoprecipitates were quantified and are presented as % of the respective lysate signal normalized for the total protein amount in the lysate.

To dissect the molecular mechanism of the effect of flotillins on FA dynamics, we made use of our yeast two-hybrid screen results, in which we identified α-actinin as an interaction partner of flotillins. During FA remodeling, α-actinin is associated with and phosphorylated by FAK and regulates its phosphorylation, thus playing an important role in the dynamics of FAs [46]. To verify the interaction of α-actinin with flotillins, we used GST pulldown experiments with a purified α-actinin-1–GST (Figure 7a). To gain insight into which flotillin preferentially interacts with α-actinin, we used MCF10A cells in which flotillins were stably knocked down by means of lentiviral shRNAs [44]. In these cells, knockdown of flotillin-1 does not significantly reduce flotillin-2 expression, but there is some variation in the residual flotillin-2 amount between experiments, resulting in broad error bars (Supplementary Figure S3). However, flotillin-2 shRNAs depletes both flotillins, reducing the amount of flotillin-1 to about 20% (Supplementary Figure S3a,b). Immunostaining of these cells showed a reduction of flotillin-1 below detection limit in all flotillin-shRNA clones and an ablation of flotillin-2 signal in the flotillin-2 shRNA clones (Supplementary Figure S3c). 

In a pulldown assay using α-actinin–GST, both flotillins were pulled down from the control cells, whereas flotillin-2 was reduced below the detection limit in the pulldown fraction upon flotillin-1 knockdown (Figure 7a, pulldown lane F1-shRNA), implicating that flotillin-1 is required for the association of flotillin-2 with α-actinin. However, a faint signal for flotillin-1 was detected in flotillin-2 shRNA cells, suggesting that flotillin-1 is capable of binding α-actinin. This signal originated from the residual fraction (20%) of flotillin-1 that was left in these cells. To verify this result with another method, endogenous α-actinin was immunoprecipitated from MCF10A cells, which endogenously express high amounts of α-actinin and flotillins (Figure 7b). Again, we used the stable MCF10A control cells and flotillin-1 knockdown cells. Endogenous flotillin-2 was found to coprecipitate with endogenous α-actinin. In flotillin-1 knockdown cells, the coprecipitation of flotillin-2 with α-actinin was very weak (6.3% and 9.1% as compared to 35% in control cells), indicating that, also in this assay, flotillin-1 appears to enhance their interaction.

### 3.7. Flotillins Colocalize and Interact with α-Actinin in Lamellipodia-Like Structures 

**Figure 8:**
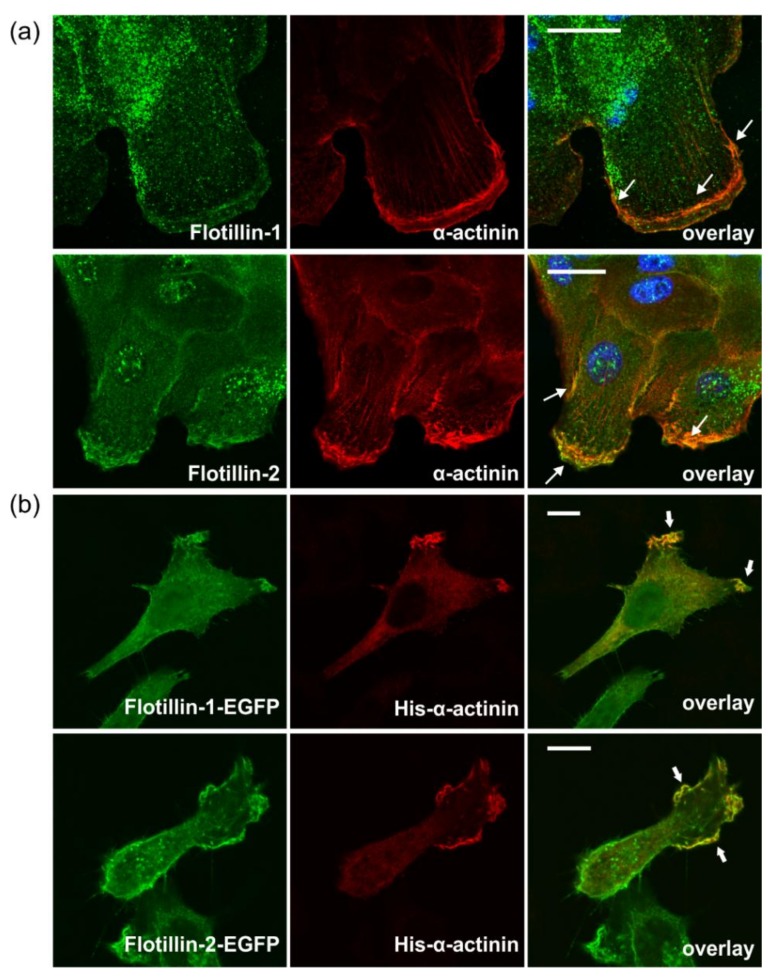
Flotillin-1 and flotillin-2 colocalize with α-actinin in peripheral membrane ruffles. (**a**) MCF10A cells were seeded on coverslips, and endogenous α-actinin and flotillin-1 (upper row) or flotillin-2 (lower row) were visualized with specific antibodies. (**b**) HeLa cells were transfected with the indicated constructs. His–α-actinin was stained with an anti-His-tag antibody. The arrows in the overlay images point to the colocalization of flotillins with α-actinin. Scale bar = 10 µm.

**Figure 9:**
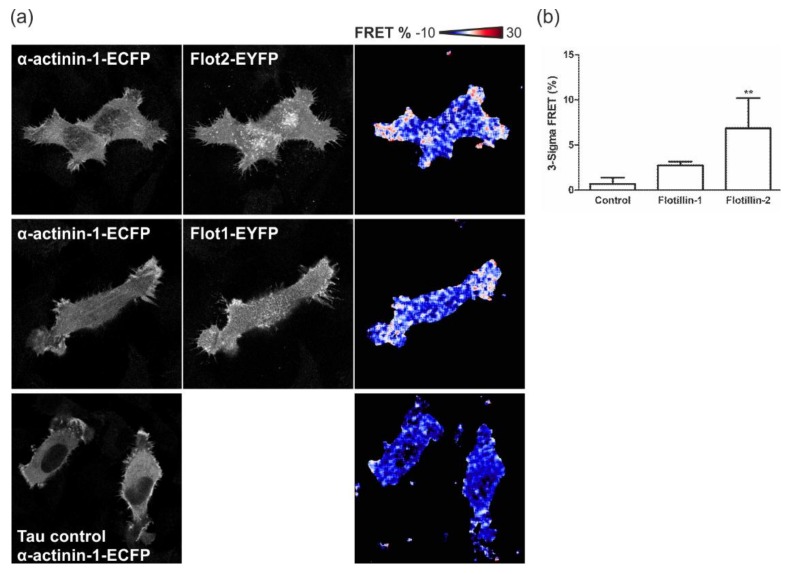
Flotillins and α-actinin-1 display Förster Resonance Energy Transfer (FRET) at the cell periphery. (**a**) FRET– Fluorescence Lifetime Imaging (FLIM) analysis was performed with HeLa cells transfected with α-actinin-1–ECFP (FRET donor) alone (lowermost row) or together with flotillin-2–EYFP (uppermost row) or flotillin-1–EYFP (middle row) as acceptors. The reduction of the fluorescence lifetime of the donor was used to calculate FRET efficiencies (see look-up-bar in the upper right corner for color coding). As compared to the τ control, an increased FRET signal is observed, indicated by the appearance of the red color. (**b**) FRET in the donor–acceptor specimen was quantified using a statistical approach. The τ distributions of all donor-only specimen were normalized and averaged. The lower 3-sigma threshold of the donor-only τ distribution was used for determining the percentage of pixels in the donor–acceptor specimen with τ lower than the threshold. The data (mean ± SD) from replicate experiments and images (*n* = 5–7) were evaluated for statistical significance using One-Way Anova. ** *p* ≤ 0.01.

In line with the above data, we also detected a considerable colocalization of endogenous flotillin-1 and flotillin-2 with α-actinin in MCF10A cells, with all proteins concentrated at the cells edges in membrane ruffle-like structures (Figure 8a, arrows). A similar colocalization was observed in HeLa cells cotransfected with His-tagged α-actinin together with flotillin-1–EGFP or flotillin-2–EGFP (Figure 8b). Since colocalization as such does not imply that two proteins also interact in the cells, we used Förster Resonance Energy Transfer (FRET) in a Fluorescence Lifetime Imaging (FLIM) setting to localize the areas of interaction of flotillins and α-actinin in the cells (Figure 9). HeLa cells were transfected with α-actinin–ECFP as the FRET donor, alone or together with either flotillin-1–EYFP or flotillin-2–EYFP as the FRET acceptor, and the fluoresence lifetime of the donor (tau, τ) was determined. A uniform tau distribution was seen in cells expressing α-actinin–ECFP alone (Figure 9a, lowermost row: tau control, and Figure 9b). Consistent with the colocalization and coprecipitation data, both flotillin-2 and flotillin-1 were found to interact with α-actinin, as indicated by the reduced τ of the donor at the peripheral regions of the cells (Figure 9a). FRET was quantified using a probability approach, where the fraction of pixels in the donor–acceptor specimen with τ lower than 99.7% of the donor-only τ was calculated and plotted as percentage of pixels (Figure 9b). A statistically significant fraction of 6.9% of the pixels in flotillin-2 and α-actinin samples exhibited τ lower than that of the donor-only control. However, in the flotillin-1 and α-actinin specimen, a non-significant fraction of 2.7% displayed these characteristics. Thus, both flotillins but especially flotillin-2, interact with α-actinin in the peripheral regions of the cells.

## 4. Discussion

In this study, we have shown that flotillins are involved in the regulation of cell migration, cell–matrix adhesion and signaling in FAs. Interestingly, siRNAs against flotillin-1 and flotillin-2 showed partially different effects on FAs. In general, we observed more profound effects upon transfection with flotillin-2 siRNAs, although a major fraction of the cellular pool of flotillins comes about as flotillin-1/flotillin-2 hetero-oligomers, and knockdown of flotillin-2 also results in a high degree of reduction of flotillin-1 expression in Hela cells. However, a reduced phosphorylation of the MAP kinase ERK2 during spreading was observed in both flotillin-1 and flotillin-2 siRNA cells, which express highly reduced amounts of flotillin-1, suggesting that the deficient ERK activation is probably more attributable to the absence of flotillin-1 than to that of flotillin-2. This is in accordance with our previous data on the role of flotillin-1 as a regulator of growth factor-dependent ERK activation and as a MAP kinase scaffolder [19]. 

One possible mechanism by which flotillins influence FAs could be through an effect on their dynamics. This is supported by the change in the FA number in steady-state cells, as well as in FA length and morphology during dynamic processes such as spreading, and by the changes in the phosphorylation of FA resident proteins such as FAK upon flotillin depletion. The actively spreading flotillin-2 siRNA cells are able to form FAs, which are, however, morphologically aberrant (elongated), pointing to a defect in the dynamic remodeling of FAs. However, we cannot say at this point if flotillin knockdown directly affects the assembly or the disassembly phase of FAs, as cell migration and spreading both require a continuous remodeling of FAs. 

A previous study has suggested a colocalization of flotillins with the cbl-associated protein (CAP) in FAs in rodent N2a neuroblastoma cells [38]. Although flotillins do not appear to be continuously localized in FAs in the cell types used in the present study, they clearly play an important role in the regulation of cell–matrix adhesion. Furthermore, a colocalization of flotillins with α-actinin was observed in peripheral membrane ruffles in MCF10A and HeLa cells. In this study, we did not observe a clear colocalization of flotillins with FA components. However, previous findings using FA proteomics have revealed that, in fibroblasts, flotillins are indeed present in FAs, and their FA localization is dependent on the activity of myosin IIa [47]. Upon myosin IIa inhibition with blebbistatin, flotillins become less associated with FAs, with flotillin-2 showing the same degree of dependency on myosin IIa activity as some well-characterized FA proteins, such as talin, vinculin, and vinexin, whereas flotillin-1 dependency is even more pronounced. Intriguingly, flotillins in turn appear to be required for the activation of myosin IIa, the activation of which is impaired in neutrophils from flotillin-1 knockout mice and upon siRNA-mediated knockdown of flotillins in HeLa cells [33]. It remains to be demonstrated if the regulation of myosin IIa activity by flotillins is dependent on their FA localization. Nevertheless, these studies together with our present data clearly point to a major role for flotillins in cell–matrix adhesion. 

In this study, we identified α-actinin as a novel direct interaction partner of flotillins using in vitro pulldowns, coimmunoprecipitation, and FRET analysis. Coprecipitation of both flotillins together with α-actinin was detected in MCF10A cells, and flotillins were pulled down by α-actinin-GST. However, reduction of flotillin-1 amount resulted in impaired coprecipitation and pulldown of flotillin-2, implicating that flotillin-1 may be important for the interaction of flotillin-2 with α-actinin. In line with this, we also observed a higher degree of FRET of α-actinin with flotillin-2 than with flotillin-1. Furthermore, siRNAs against flotillin-2 (increase) and flotillin-1 (decrease) resulted in opposite changes in the length of FAs, and only flotillin-2 siRNAs impaired FAK phosphorylation, whereas flotillin-1 siRNAs did not produce any detectable changes in FAK phosphorylation. 

The role of flotillins during neurite outgrowth and differentiation of neuronal cells, which involve cytoskeletal remodeling and FA dynamics, has previously been addressed by some studies. In N2a cells that expressed a mutant form of flotillin-2 consisting of its C-terminal part (R1EA), the activation of the Rho family GTPases, which are important mediators of actin remodeling, was impaired [38]. Upon insulin-like growth factor (IGF) stimulation, cdc42 showed an increased basal activation, whereas Rac1 activity did not exhibit the normal increase upon IGF stimulation. Furthermore, FAK activation (measured as phosphorylation on Tyr residues 567 and 577) was enhanced, whereas no effect could be seen on the activation of ERK2, protein kinase B (PKB), or protein kinase C (PKC). Later on, the same group showed that downregulation of flotillin-2 expression resulted in impaired neurite outgrowth in N2a cells. In flotillin-2 knockdown cells, Rac1 was shown to exhibit an increased basal and IGF-induced activation, whereas cdc42 activity was reduced and not affected by IGF [39]. In addition, a decreased phosphorylation of p38 and FAK on Y567/577 was detected, with concomitant decrease in the levels of the total FAK protein. ERK2 activity was moderately diminished, whereas no effect could be seen on PKB and PKC [39]. Although these results show that flotillin-2 depletion can affect the activation of FA proteins, they are not directly comparable with those presented here, since the authors used growth factor stimulation in firmly adhered cells, whereas we have measured the activation during active cell spreading. However, it appears that the regulatory function of flotillins on FAs is conserved among different cell types and during various processes requiring FA remodeling, such as neurite outgrowth [38,39] and cell migration and spreading (this paper). 

How do flotillins then affect the dynamics of FAs and the phosphorylation status and localization of FA proteins? One possibility is that flotillins regulate FA turnover by influencing the formation of signaling complexes that are required for this process. As mentioned above, α-actinin and FAK cooperate during FA remodeling. Y397-phosphorylated, active FAK binds the Src kinase, which facilitates the phosphorylation of further Tyr residues of FAK, which then phosphorylates α-actinin at Tyr12. The phosphorylation of α-actinin at Y12 creates a binding site for Src. Upon Src association with pY12 α-actinin, the complex between FAK and c-Src is disrupted. This exposes FAK to dephosphorylation by the phosphatase PTB-1B, which in turn facilitates FA turnover. Thus, by influencing the localization of α-actinin and FAK, flotillins might be able to regulate the accessibility of the signaling partners to each other, and hence also FA turnover. Consistent with this model, we here detected changes both in the phosphorylation status of FAK and in the morphology of FAs using FAK and α-actinin staining in flotillin knockdown cells. Furthermore, we have previously shown that Src-mediated phosphorylation of flotillin-2 on Tyr163 is important for cell spreading on fibronectin [21] and might thus also affect complex formation between FA regulatory proteins. On the other hand, the localization of α-actinin in FAs appears to be dependent on myosin IIa activity [47], which in turn may depend on flotillins [33]. Thus, although flotillins and α-actinin also directly interact, the observed changes in FA morphology and α-actinin in flotillin2 knockdown cells might also be partly due to the decreased myosin IIa activity in the cells. 

Flotillin overexpression has been clearly associated with various types of cancers such as breast cancer and melanoma, and increased flotillin expression frequently correlates with poor prognosis and reduced survival (reviewed in [48]). Studies on flotillin-2 knockout mice interbred with a breast cancer mouse model, MMTV-PyMT (mouse mammary tumor virus-polyoma middle T antigen) mice, showed that flotillins might be important especially for the formation of metastases [32]. Although no effect on primary tumor formation could be detected in the absence of flotillin expression, the formation of lung metastases was significantly reduced [32]. Interestingly, flotillin-1 translation is controlled by the micro-RNA *miR-124*, which is frequently downregulated in breast tumors, correlating with increased flotillin-1 expression [49]. Cell–matrix adhesion and cell migration are vitally important for metastasis formation and cancer spreading. Thus, the findings of the present study together with numerous studies linking flotillins to cancer point to a possible role of flotillins in metastasis formation by means of regulating cell–matrix contacts. Shedding light on this and identifying the exact molecular mechanisms will be an important aspect of future studies on flotillin function during cell–matrix adhesion. 

## Supplementary Materials

The following are available online at http://www.mdpi.com/2073-4409/7/4/28/s1, Figure S1: Phenotype of flotillin knockdown cells; Figure S2: Flotillin knockdown cells show fewer focal adhesions; Figure S3: Stable knockdown of flotillin-1 or flotillin-2 in MCF10A cells.
