# Supplementary material for: Flotillins Regulate Focal Adhesions by Interacting with α-Actinin and by Influencing the Activation of Focal Adhesion Kinase
Source: Cells. 2018 Apr 7;7(4):28. doi: 10.3390/cells7040028 (PMC5946105; doi:10.3390/cells7040028)
Supplement: Supplementary file 1 [file cells-07-00028-s001.pdf]

Supplementary Figure S1, Banning et al.

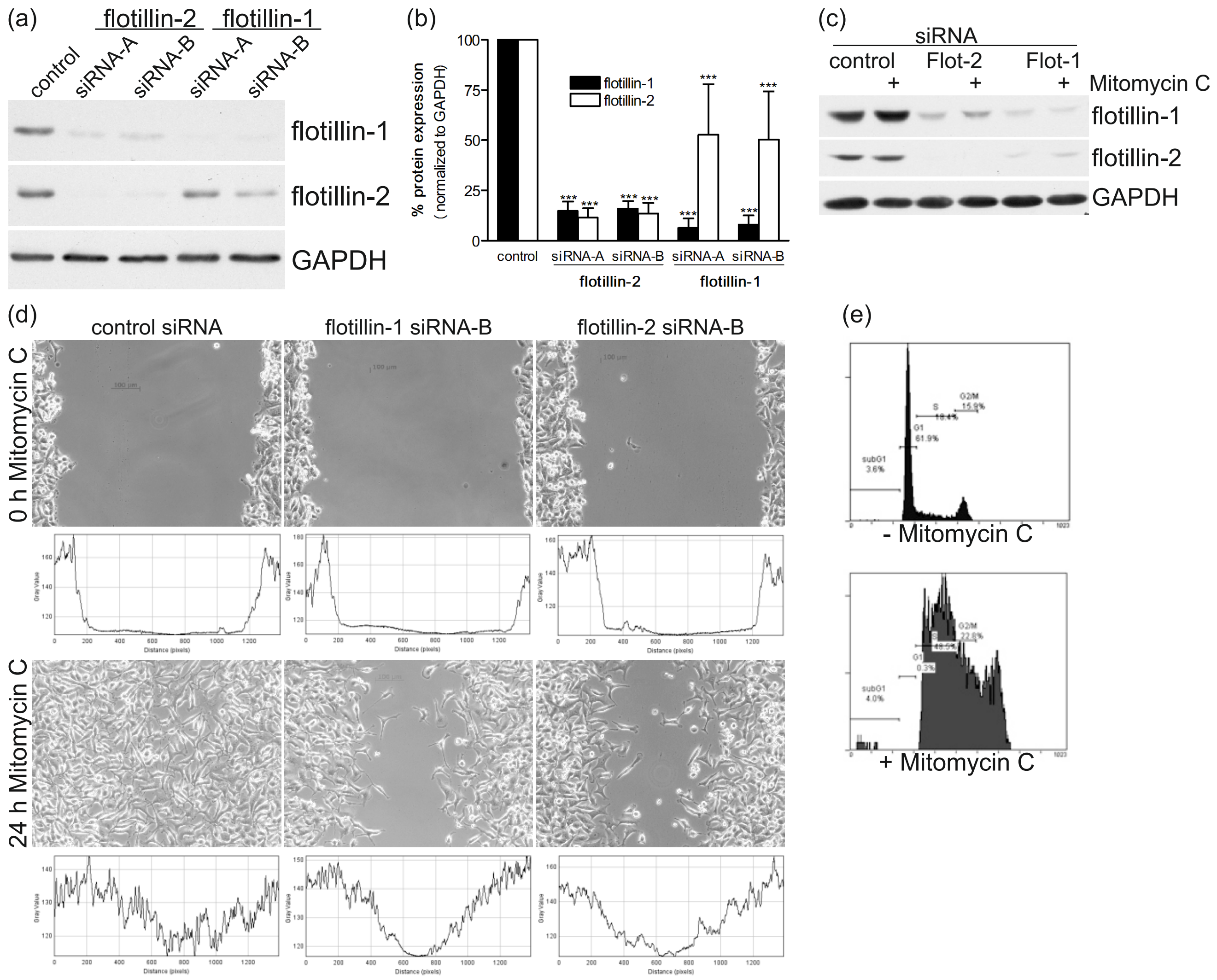


**Supplementary Figure S1. Phenotype of flotillin knockdown cells**. (a) Western blot detection of flotillins in HeLa cells transfected with the indicated siRNAs. (b) quantification of the transient flotillin knockdown in HeLa cells and its effect on flotillin protein expression. Bars show mean ± SD (n=4, ***p<0.001, One-way ANOVA). (c) Transient knockdown of flotillins in HeLa cells used for the wound healing was controlled by Western blot. (d) HeLa cells were transfected with the indicated siRNAs and allowed to grow into confluency. A defined scratch was then produced (0 h, upper panels), and the closure of the wounded area was monitored over 24 h (lower panels) during which the cells were grown under Mitomycin C to prevent proliferation. (e) The effect of Mitomycin C on the cell cycle was controlled using propidium iodide staining and fluorescence-assisted cell sorting.

Supplementary Figure S2, Banning et al.

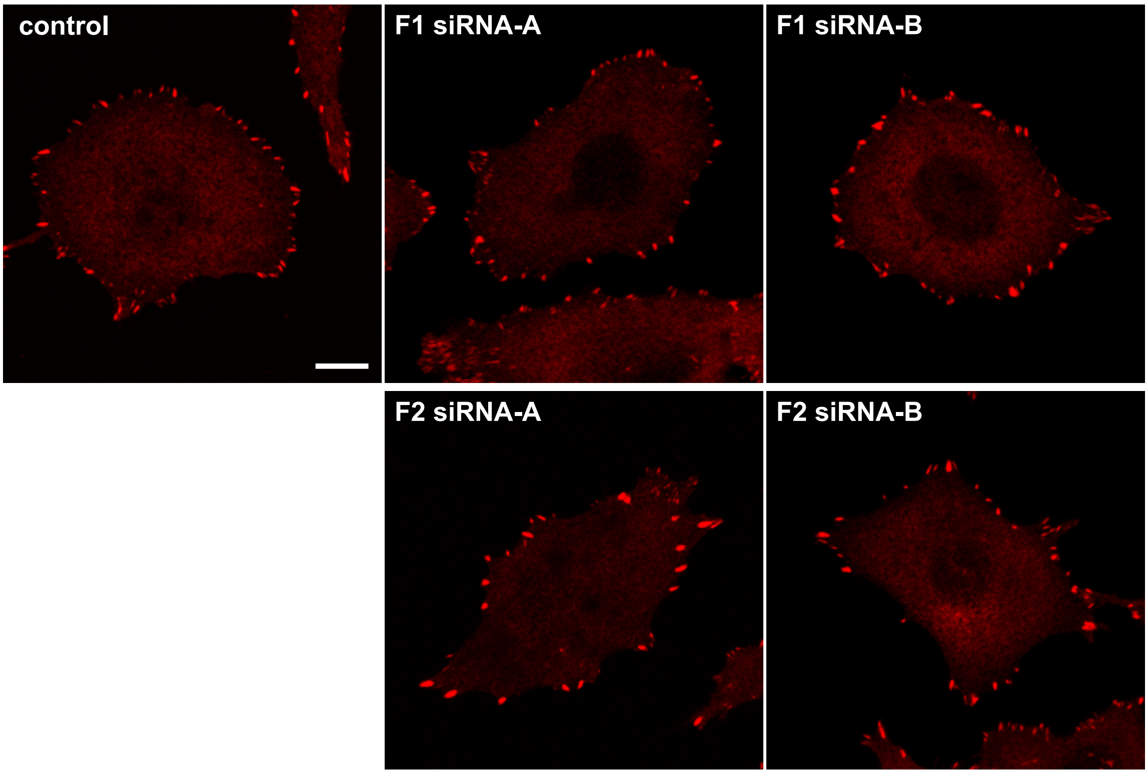


**Supplementary Figure S2. Flotillin knockdown cells show fewer focal adhesions.** HeLa cells were transfected with the indicated siRNAs, and focal adhesions were visualized by vinculin staining. Scale bar: 10 µm, the same magnification for all images.Quantification of the data is shown in Fig. 1d.

# Supplementary Figure S3, Banning et al.

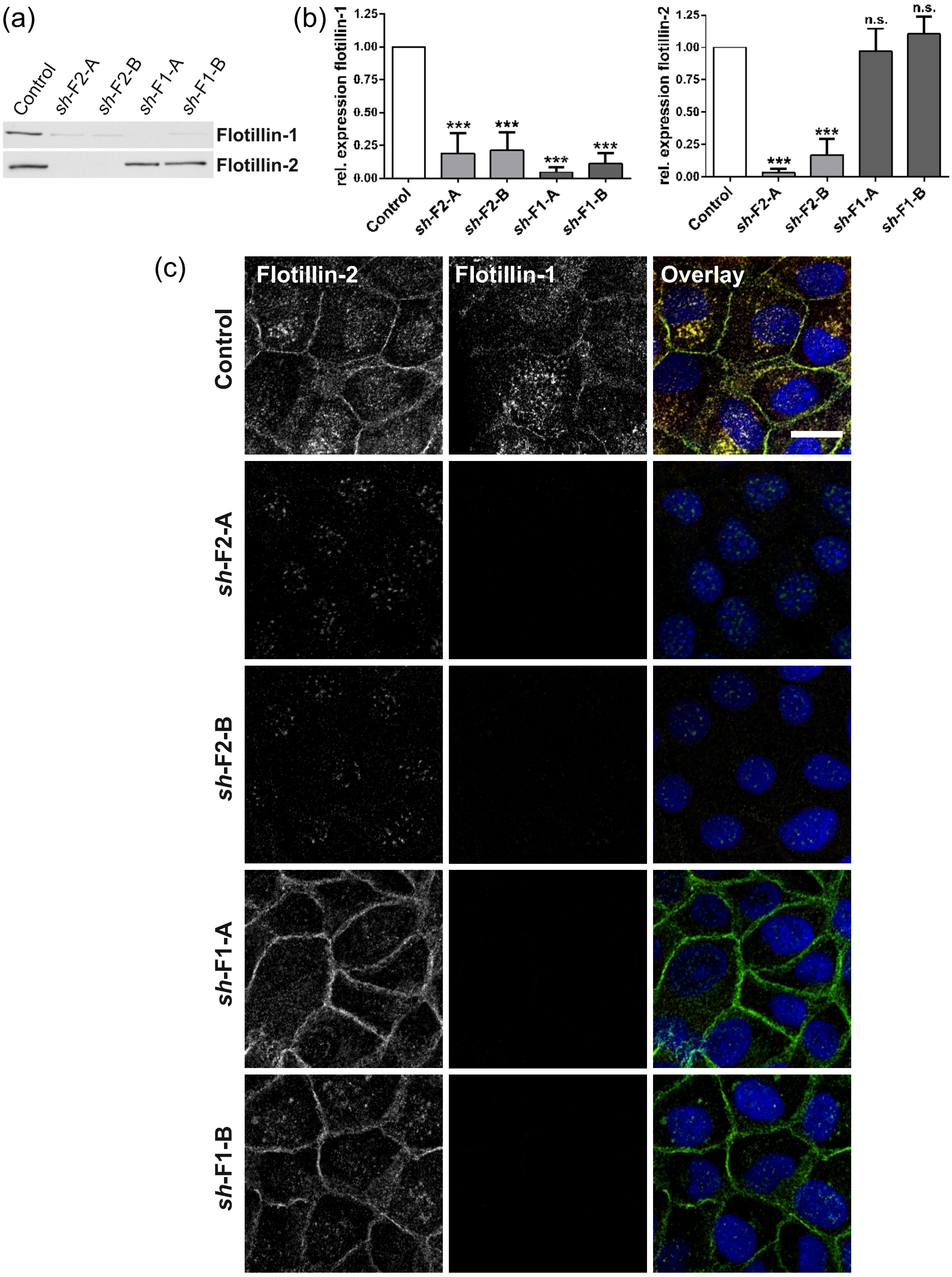


**Expression and localization of flotillins in stable MCF10A knockdown cells.** (a) Expression of flotillins in MCF10A cells stably transfected with shRNAs against flotillin-2 (*sh*-F2-A/B) or flotillin-1 (*sh*-F1-A/B). (b) Densitometric quantification of flotillin-2 and flotillin-1. The signals were normalized to GAPDH. Bars represent the mean ± S.D. of three independent experiments. One-way ANOVA with Bonferroni's multiple comparison test. ***, $p < 0.001$; n.s., not significant. (c) Staining of flotillin-1 and flotillin-2 in stable MCF10A knockdown cells. Scale bar: 20 µm.
